# Against mass media trends: Minority growth in cultural globalization

**DOI:** 10.1371/journal.pone.0230923

**Published:** 2020-04-02

**Authors:** M. G. Cosenza, M. E. Gavidia, J. C. González-Avella

**Affiliations:** 1 School of Physical Sciences & Nanotechnology, Universidad Yachay Tech, Urcuquí, Ecuador; 2 Luxembourg Centre for Systems Biomedicine, University of Luxembourg, Belvaux, Luxembourg; 3 APSL, Palma de Mallorca, Spain; 4 Universidad de Los Andes, Mérida, Venezuela; 5 Institute for Cross-Disciplinary Physics and Complex Systems, IFISC, UIB-CSIC, Palma de Mallorca, Spain; Centre National de la Recherche Scientifique, FRANCE

## Abstract

We investigate the collective behavior of a globalized society under the influence of endogenous mass media trends. The mass media trend is a global field corresponding to the statistical mode of the states of the agents in the system. The interaction dynamics is based on Axelrod’s rules for the dissemination of culture. We find situations where the largest minority group, possessing a cultural state different from that of the predominant trend transmitted by the mass media, can grow to almost half of the size of the population. We show that this phenomenon occurs when a critical number of long-range connections are present in the underlying network of interactions. We have numerically characterized four phases on the space of parameters of the system: an ordered phase; a semi-ordered phase where almost half of the population consists of the largest minority in a state different from that of the mass media; a disordered phase; and a chimera-like phase where one large domain coexists with many very small domains.

## 1 Introduction

In a social context, globalization implies increasing interconnectivity among people: any agent may interact with any other in a social system. Internet, social networks, mobile phones, and other technologies have contributed to the realization of fully connected societies. In recent years, the globalization of culture has been an issue of much interest in Social Sciences [1]. It generally refers to the exchange of cultural symbols among people around the world that leads to the formation of shared norms and behaviors with which people associate their individual identities and collective culture [2–4]. Although there is no consensus on the consequences of globalization on local cultures, many scholars sustain that the exposure of minority groups to a global culture can undermine their own cultural identity [5–8]. One of the most controversial debates on the matter of cultural globalization and the “minority identity crisis” involves the role of mass media as a facilitating tool for its expansion or limitation [9–12].

With the advent of globalization, broadcasting, telecommunication, and internet-based companies have also become global; mass media messages can practically reach all individuals and groups in the society. The interaction with mass media have experienced important changes: in on one hand people get informed by the media, while on the other hand the information is influenced by the evolution of people’s preferences [13–15]. To some extent, main stream media adapt their contents to reflect the predominant tendencies, fashionable behavior, and cultural trends in the globalized society [16]. Mass media trends represent a plurality information feedback in social systems with endogenous cultural influences [17–19]. Global information feedback also occurs, for example, in virtual platforms, such as Amazon, where users have access to the average valuation of a commercial product resulting from their own opinions.

Models of mass media acting on social systems have been shown to produce counterintuitive effects: cultural diversity increases when the intensity of the mass media message is strong enough, whereas weaker messages impose their state leading to global cultural homogeneity [20–24].

In this article we investigate the collective behavior of a globalized society under the influence of endogenous mass media trends. As a model for this system, we consider a fully connected network of social agents subject to a global field representing the mass media trend. In particular, we study the evolution of minority groups in presence of the predominant cultural trends transmitted by the media. We employ Axelrod’s rules for the dissemination of culture as interaction dynamics [25], a non-equilibrium model of wide interest for physicists [20–24, 26–32]. We find situations where the predominant tendencies of mass media are not followed by the entire population. We uncover a nontrivial minority growth phenomenon induced by the endogenous mass media for some values of parameters: a minority group possessing a cultural state alternative to that of the mass media trend can increase its size up to almost half of the size of the population. We show that the growth of the largest minority group induced by mass media trends on a social system requires two features: an endogeneous global field and the presence of global or long-range interactions. Previous models of mass media influence do not generally include the coexistence of both features. Additionally, for some conditions the system segregates into two distinguishable subsets: agents in one subset share the state of the mass media trend, while agents in the other subset exhibit a disordered or incoherent state. This configuration is analogous to a chimera state arising in networks of globally coupled oscillators.

In Sec. 2 we present the model for a globally connected network of social agents subject to mass media trends. In Sec. 3 we describe the growth of a minority and characterize the collective states arising on the space of parameters of the system. The dynamics of the minority growth phenomenon is also investigated. Section 4 contains our conclusions.

## 2 Materials and methods

### Model for mass media trends in a globally connected network

We consider a population of *N* social agents consisting of a fully connected network, where every agent can interact with any other in the system. We assume that interactions take place according to the dynamics of the model of cultural dissemination of Axelrod [25]. The state variable of agent *i* (*i* = 1, 2, …, *N*) is given by the *F*-component vector $x_{i}=\left(x_{i}^{1},\ldots ,x_{i}^{f},\ldots ,x_{i}^{F}\right)$, where each component $x_{i}^{f}$ represents a cultural feature that can take any of *q* different traits or options in the set {0, 1, …, *q* − 1}. In this paper we employ the normalized parameter *Q* = 1 − (1 − 1/*q*)^*F*^ to represent the decreasing number of options per feature, such that *Q* = 0 for *q* → ∞ (large number of options) and *Q* = 1 for *q* = 1 (one option). We denote by *M* = (*μ*^1^, …, *μ*^*f*^, …, *μ*^*F*^) the global field defined as the statistical mode of the states in the system at a given time. Then, the component *μ*^*f*^ corresponds to the most abundant value shown by the component $x_{i}^{f}$ of all the state vectors *x*_*i*_ in the population. If the most abundant value is not unique, one of the possibilities is chosen at random with equal probability. We assume that each agent is subject to the influence of the global field *M*. In the context of cultural models, this global field represents an endogeneous mass media influence acting on all the agents in the system and containing the most predominant trait in each cultural feature present in the population; i. e., a global cultural trend or fashionable behavior.

In this paper we fix the parameter value *F* = 10. Initially, the states *x*_*i*_ are assigned at random with a uniform distribution. At any given time, a randomly selected agent can interact either with any other agent in the system or with the global field *M*, according to the rules of Axelrod’s cultural model. We define the dynamics of the system by the following iterative algorithm:

1. Select at random an agent *i*, called *active agent*.
2. Select the *source of interaction*: with probability *B* active agent *i* chooses to interact with the field *M*, while with probability (1 − *B*) it chooses to interact with another agent *j* taken at random.
3. Calculate the overlap between the active agent and the source of interaction, defined as the number of shared components between their respective vector states, $l\left(i,y\right)=\sum_{f=1}^{F}\delta_{x_{i}^{f},y^{f}}$, where *y*^*f*^ = *μ*^*f*^ if the source of interaction is the field *M*, or $y^{f}=x_{j}^{f}$ if *i* interacts with *j*. We use the delta Kronecker function: *δ*_*x*,*y*_ = 1, if *x* = *y*; *δ*_*x*,*y*_ = 0, if *x* ≠ *y*.
4. If 0 < *l*(*i*, *y*) < *F*, with probability *l*(*i*, *y*)/*F* active agent *i* interacts with the selected source of interaction, as follows: choose *h* at random such that $x_{i}^{h}\neq y^{h}$ and set $x_{i}^{h}=y^{h}$. If *l*(*i*, *y*) = 0 or *l*(*i*, *y*) = *F*, the state of agent *i* does not change.
5. If the source of interaction is *M*, update the field *M*.

The parameter *B* ∈ [0, 1] in step (2) describes the probability of the global cultural trend to influence all agents in the system and represents the intensity of the field *M*. Steps (3) and (4) describe the interaction rules from Axelrod’s model. Step (5) characterizes the time scale for the updating of the global field *M*. It expresses the assumption that agents do not have instantaneous information about the mass media trend, but only when they effectively interact with the media. As the intensity *B* of the global field increases, the updating rate of the state of the global field also increases.

In the absence of mass media influence (*B* = 0), a system subject to Axelrod’s dynamics reaches an asymptotic state in any finite network where domains of different sizes are formed. A domain is a set of connected agents that share the same state. A homogeneous collective state or ordered phase in the system is characterized by having overlap *l*(*i*, *j*) = *F*, ∀*i*, *j*. This state possesses *q*^*F*^ equivalent realizations. In an inhomogeneous collective state or disordered phase, several domains coexist. It has been found that, on several networks, the system reaches an ordered phase for values *q* < *q*_*c*_, and a disordered phase for *q* > *q*_*c*_, where *q*_*c*_ is a critical point [26–28, 33]. In terms of the normalized parameter *Q*, the disordered phase occurs for *Q* < *Q*_*c*_ = 1 − (1 − 1/*q*_*c*_)^*F*^ and the ordered phase arises for *Q* > *Q*_*c*_.

To characterize the collective behavior of the system under the influence of global mass media trends, we employ two statistical quantities as order parameters: (i) the average normalized size (divided by *N*) of the largest domain, denoted by *S*_1_; and (ii) the average normalized size of the second largest domain, assigned as *S*_2_. Thus, in the absence of mass media influence (*B* = 0), an ordered phase for values *Q* > *Q*_*c*_ is characterized by *S*_1_ → 1, while a disordered phase for *Q* < *Q*_*c*_ has *S*_1_ → 0.

## 3 Results

### Emergence of minorities and chimera states

**Fig 1:**
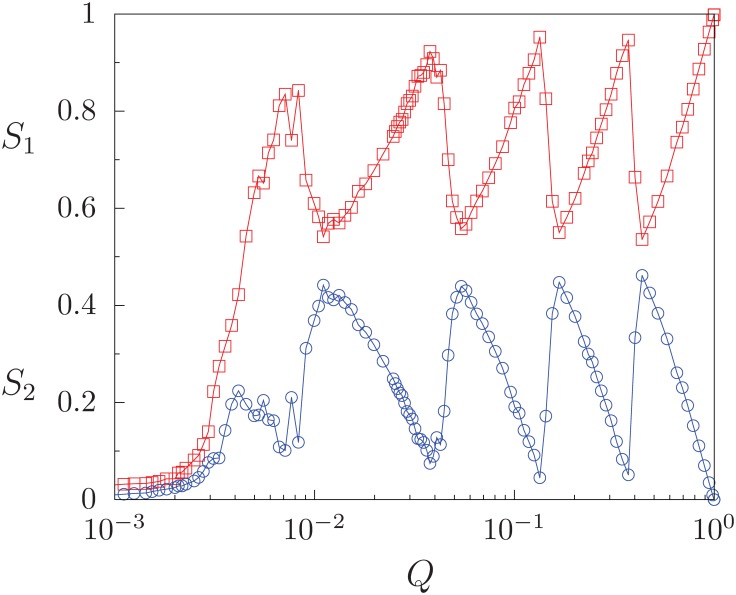
Order parameters *S*_1_ (squares) and *S*_2_ (circles) as functions of *Q* (log scale) for *B* = 0.8. Each data point is the result of averaging over 100 realizations of initial conditions. Fixed parameters are *F* = 10, *N* = 800.

As the interaction probability *B* increases from zero, the behavior of the quantity *S*_1_ notably changes. In Fig 1 we show both order parameters *S*_1_ and *S*_2_ as functions of the normalized number of options *Q* for a fixed value of *B* > 0. The quantity *S*_1_ exhibits several local minima with *S*_1_ < 1 at some values *Q* > *Q*_*c*_; we have found that both these values and the number of minima depend on *B*. For values *Q* < *Q*_*c*_, the system settles into a disordered state where *S*_1_ → 0.

The state of the largest domain always corresponds to the state of the field *M* that represents the predominant cultural trend. The presence of local minima in *S*_1_ indicates that, for some values of parameters, the cultural trend is not adopted by all the agents in the population. Fig 1 shows that the local minima of *S*_1_ are associated to local maximum values of *S*_2_, such that *S*_1_ + *S*_2_ ≈ 1 for *Q* > *Q*_*c*_. Therefore, two large domains that occupy almost all the system can arise for *Q* > *Q*_*c*_. The state of the largest domain is equal to that of the field *M*, but the second largest domain reaches a state different from *M*. Thus, the values of the normalized number of options *Q* for which *S*_1_ displays local minima are related to the emergence of the largest minority group ordered in a state alternative (non-interacting) to that being imposed by the mass media trend.

**Fig 2:**
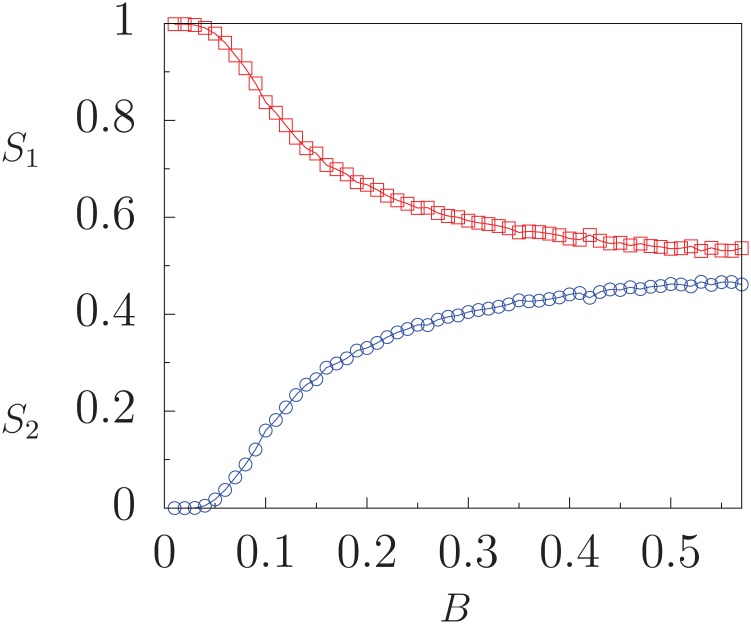
Quantities *S*_1_ (squares) and *S*_2_ (circles) as functions of the interaction probability *B*, with fixed *Q* = 0.40 (*q* = 20). Each data point is the result of averaging over 100 realizations of initial conditions. Fixed parameters: *F* = 10, *N* = 800.

To investigate the influence of the mass media cultural trend on the behavior of the largest minority in the population, in Fig 2 we show both quantities *S*_1_ and *S*_2_ as functions of the interaction probability *B* for a fixed value of parameter *Q* > *Q*_*c*_ where a local minimum of *S*_1_ appears. For small values of *B*, the largest domain makes up all the system, and thus *S*_1_ = 1, *S*_2_ = 0. As *B* increases, a competition takes place between the spontaneous order emerging in the system due to the agent-agent interactions and the order being imposed by the endogenous global field. As a consequence, a second largest domain emerges and grows its size while the largest domain decreases its size, such that both constitute almost all the population, *S*_1_ + *S*_2_ ≃ 1. Thus, we have the counter-intuitive result that, for some values of *Q* that represent the number of options per cultural feature, an increase in the intensity of the mass media message transmitting the predominant cultural trend can actually promote the growth of the largest minority group. This group can become almost half of the population size in a state different to that of the media. We denote this situation as a minority-growth state.

For greater values of *B*, the interaction of the mass media field with the agents dominates, and *S*_1_ may increase. The case *B* = 1 describes a situation where the agents solely interact with the global field *M*, which becomes initially established and then remains fixed. This scenario is indistinguishable from that of a system subject to a fixed external global field [23]. Then, the fraction of agents whose states converge to the state of the field is the fraction of agents that initially share at least one component with the field and it is given by [23]

$$\begin{array}{c} S_{1}\left(B=1\right)=1-{\left(1-1/q\right)}^{F}=Q. \end{array}$$ (1)

**Fig 3:**
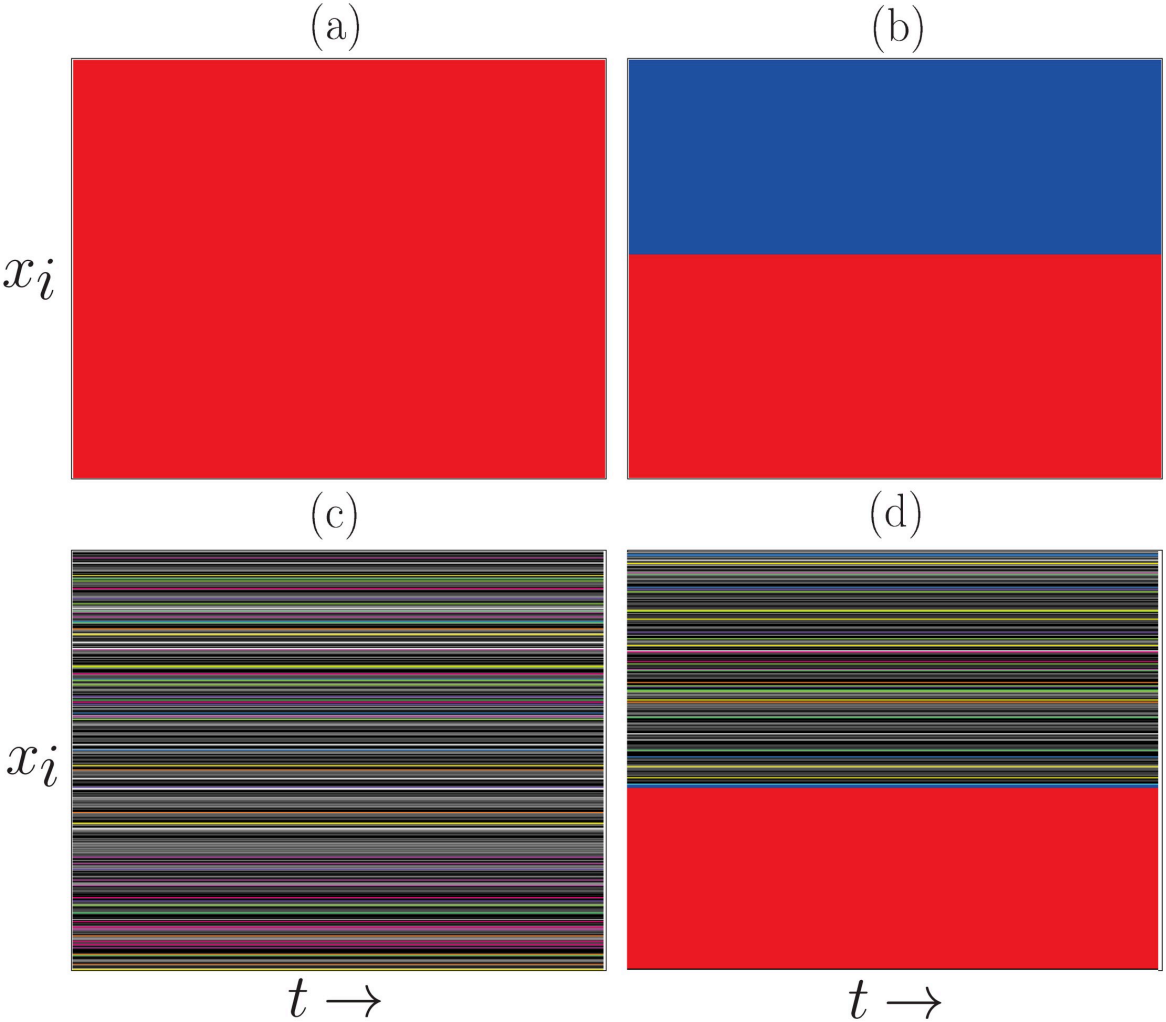
Asymptotic states *x*_*i*_ (*i* = 1, …, *N*) of the agents (vertical axis) as a function of time (horizontal axis) over 500 consecutive time steps, after discarding 3000 transients time steps, for different values of the probability *B* and the normalized number of options *Q*. Each state variable of an agent is represented by a different color; if *x*_*i*_ = *x*_*j*_, then agents *i* and *j* share the same color. Each panel displays the domains formed in the system as a function of asymptotic time. Largest domain of size *S*_1_ is represented in red; second largest domain of size *S*_2_ is assigned blue. Fixed parameters: *F* = 10, *N* = 800. (a) *B* = 0.1, *Q* = 0.79 (homogeneous state, phase I); (b) *B* = 0.9, *Q* = 0.01 (minority-growth state, phase II); (c) *B* = 0.03, *Q* = 0.0019 (disordered state, phase III); (d) *B* = 0.48, *Q* = 0.0037 (chimera state, phase IV).

In addition to the above described collective states, for some values of the interaction probability *B* and *Q* > *Q*_*c*_, the system can reach a partially coherent configuration where one part of the population forms a domain in a homogeneous state equal to *M*, while the remaining part is in a disordered state. This state is characterized by *S*_1_ > 0 and *S*_2_ → 0; the size of *S*_1_ depends on initial conditions. This situation is similar to a chimera state occurring in dynamical systems subject to global interactions, where the symmetry of the system is spontaneously broken into two coexisting coherent (or synchronized) and incoherent (or desynchronized) subsets [34–38]. Chimera states where initially identified in systems of nonlocally coupled oscillators [39, 40]. Fig 3 displays the asymptotic spatiotemporal patterns corresponding to the collective behaviors observed in the system subject to the influence of the global mass media trend, for different values of parameters *B* and *Q*.

**Fig 4:**
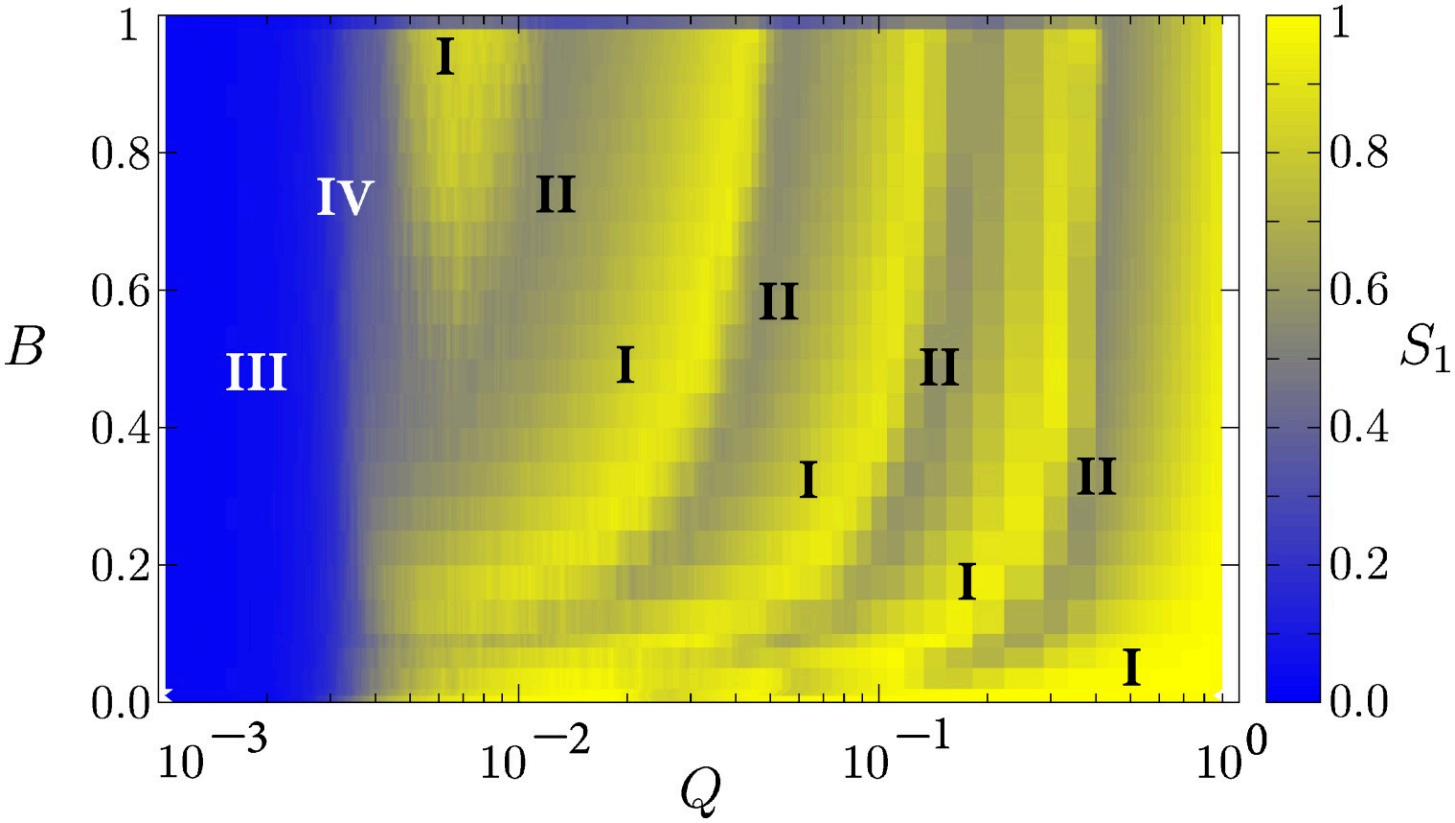
Phase diagram for the system on the space of parameters (*B*, *Q*). The color code represents the value of the order parameter *S*_1_. The regions where the different phases occur are labeled: phase I, homogeneous state; phase II, minority-growth state; phase III, disordered state; phase IV, chimera-like states. Fixed parameters are *F* = 10, *N* = 800. Each data point is averaged over 100 realizations of initial conditions.

The collective behavior of the system can be characterized on the space of parameters (*B*, *Q*), as shown in Fig 4. Four phases can be found: (I) a homogeneous, ordered phase for which *S*_1_ → 1; (II) a semi-ordered phase, where *S*_2_ > 0 and *S*_1_ + *S*_2_ ≃ 1, characterized by the growth of a second largest domain ordered in a state different from that of the mass media; (III) a disordered phase for *Q* < *Q*_*c*_, for which *S*_1_ → 0; and (IV) chimera states, characterized by *S*_1_ > 0 and *S*_2_ → 0, where one large domain coexists with many domains of negligible sizes. The chimera states depend on initial conditions, and they can emerge in regions of parameters that lie between phase I and phase III states. In fact, chimera states share features of both phase I and phase III; they can be considered as transition configurations between these two phases.

### Dynamics of minority growth

**Fig 5:**
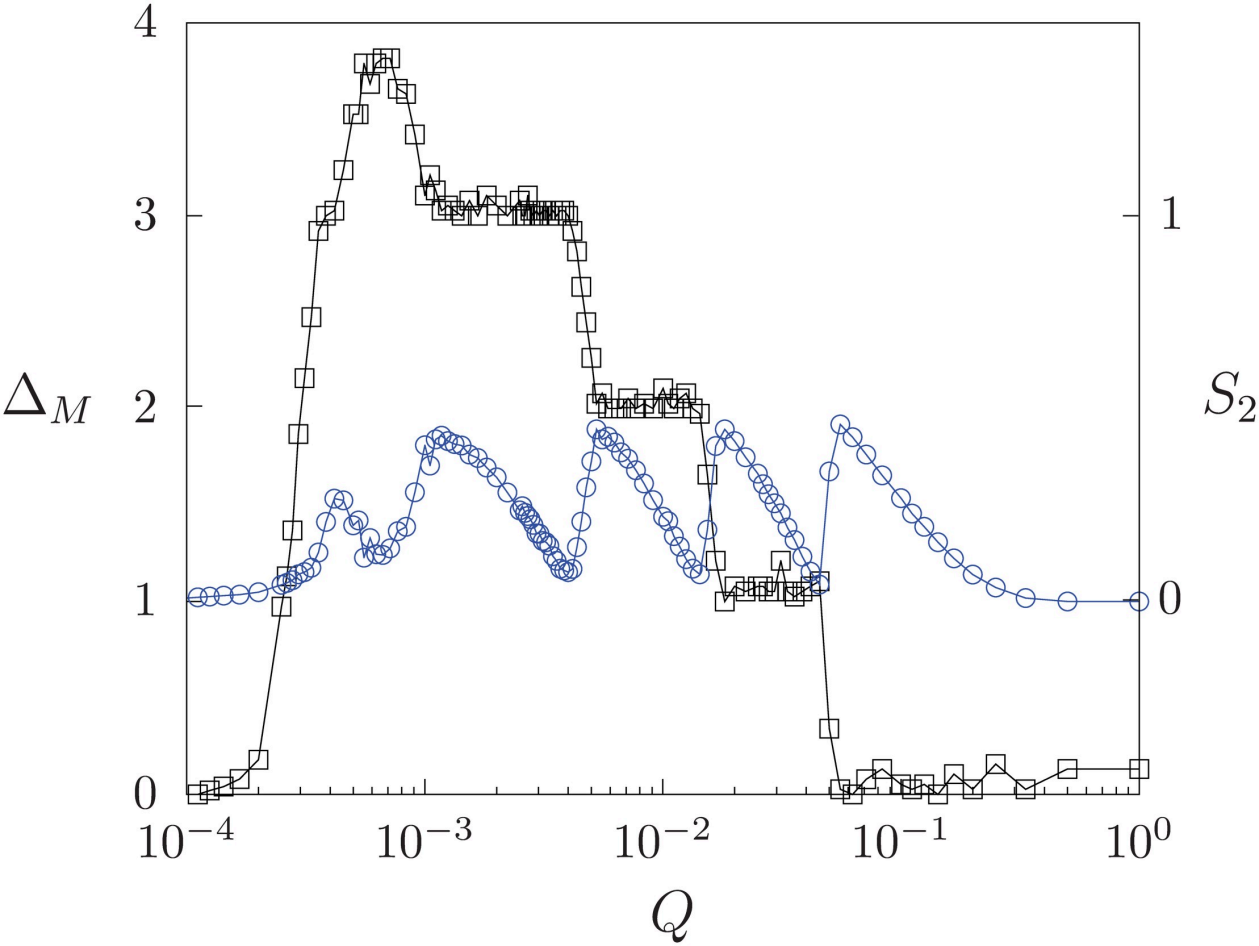
Average number of updates Δ*M* of the mass media vector (squares) as a function of the normalized number of options *Q* (log scale), with fixed *B* = 0.8. Each value Δ*M* is averaged over 50 realizations of initial conditions. For reference, the graph *S*_2_ versus *Q* (circles) with *B* = 0.8 is included without vertical scale. Fixed parameters *F* = 10, *N* = 800.

As the system evolves from its initial conditions for given parameter values, the mass media vector *M* changes until it reaches a stationary state. In Fig 5 we calculate the average number of updates or changes Δ*M* occurred to vector *M* as a function of *Q*, for a fixed value of the intensity *B* = 0.8. For comparison, the curve *S*_2_ (right vertical scale) versus *Q* for this value of *B* from Fig 1 is also included.

For values *Q* < *Q*_*c*_, there is a large number of domains and vector *M* adopts the state of any of them with equal probability since these states are equally abundant. Then, *M* does not change, and Δ*M* = 0. At the critical point *Q* = *Q*_*c*_ there occur many fluctuations in the number of domains and their sizes. As a consequence, vector *M* experiences several changes and Δ*M* suddenly increases to a value of almost 4. Since there is a probability 1 − *B* that all agents interact among themselves to form domains, the order emerging from the agent-agent interactions competes with the order being imposed by *M* for *Q* > *Q*_*c*_. A change in *M* represents a reset of initial conditions, and thus is equivalent to a change in the number of agents that can interact with the updated vector *M*, a number reflected in the size *S*_1_. The competition between the agent-field and agent-agent interactions takes place over a range of values of *Q* where the number of changes Δ*M* remains constant, allowing for the increase of *S*_1_ and a decrease of *S*_2_. Increasing the number of options represented by *Q* implies less available states and a reduction in the number of changes Δ*M*. A drop in Δ*M* represents a decrease in *S*_1_ and gives chance to the rise of the minority size *S*_2_. These processes are repeated on a sequence of intervals of *Q*, until this parameter approaches the value *Q* = 1 where there are few states available and the global field does not change, Δ*M* = 0; the agents mostly interact with a fixed global field *M* that imposes its state on them. Thus, the interplay between the global coupling among the agents and the adaptive dynamics of the autonomous global field *M* is responsible for the repeated occurrence of minority growth in a state alternative to that of the field as *Q* is varied.

**Fig 6:**
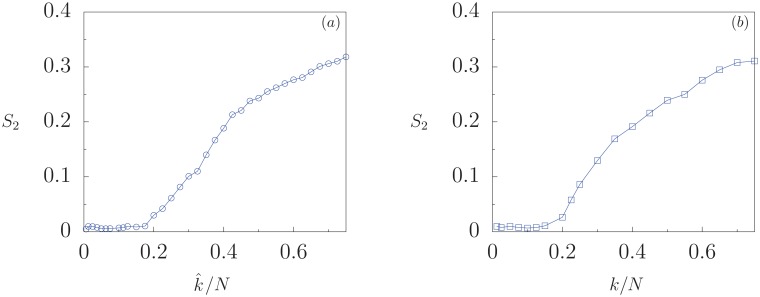
(a) Size of the second largest domain *S*_2_ as a function of the average number of neighbors $\bar{k}/N$ for the system defined on a random network. (b) *S*_2_ as a function of the number of neighbors *k*/*N* for the system defined on a ring with long range interactions. Fixed parameters in both cases are *Q* = 0.247, *B* = 0.01, *N* = 800.

To elucidate the role of the connectivity of the network on the minority growth phenomenon, we consider the dynamics of the system defined on three different topologies: (i) an Erdös-Renyi random network of *N* nodes having average degree $\bar{k}$; and (ii) a ring of *N* nodes where each node is connected to *k* neighbors, *k*/2 on each side; and (iii) a Watts-Strogatz small-world network consisting of an initial ring of *N* nodes, each with *k* neighbors, and rewiring probability *p*. The fully connected network studied above corresponds to the values $\bar{k}=N-1$ and *k* = *N* − 1, respectively. Fig 6(a) shows the size of the second largest domain *S*_2_ as a function of $\bar{k}/N$ for random networks, with fixed values of the interaction probability *B* and *Q* > *Q*_*c*_. When $\bar{k}$ is small, the system reaches a homogeneous state with *S*_2_ = 0 and *S*_1_ = 1. However, *S*_2_ grows as the average number of neighbors increases above a threshold value $\bar{k}/N\approx 0.22$. Similarly, Fig 6(b) shows *S*_2_ as a function of *k*/*N* for a ring-type network. The size *S*_2_ increases from zero above some threshold value of the fraction of neighbors *k*/*N* ≈ 0.22.

**Fig 7:**
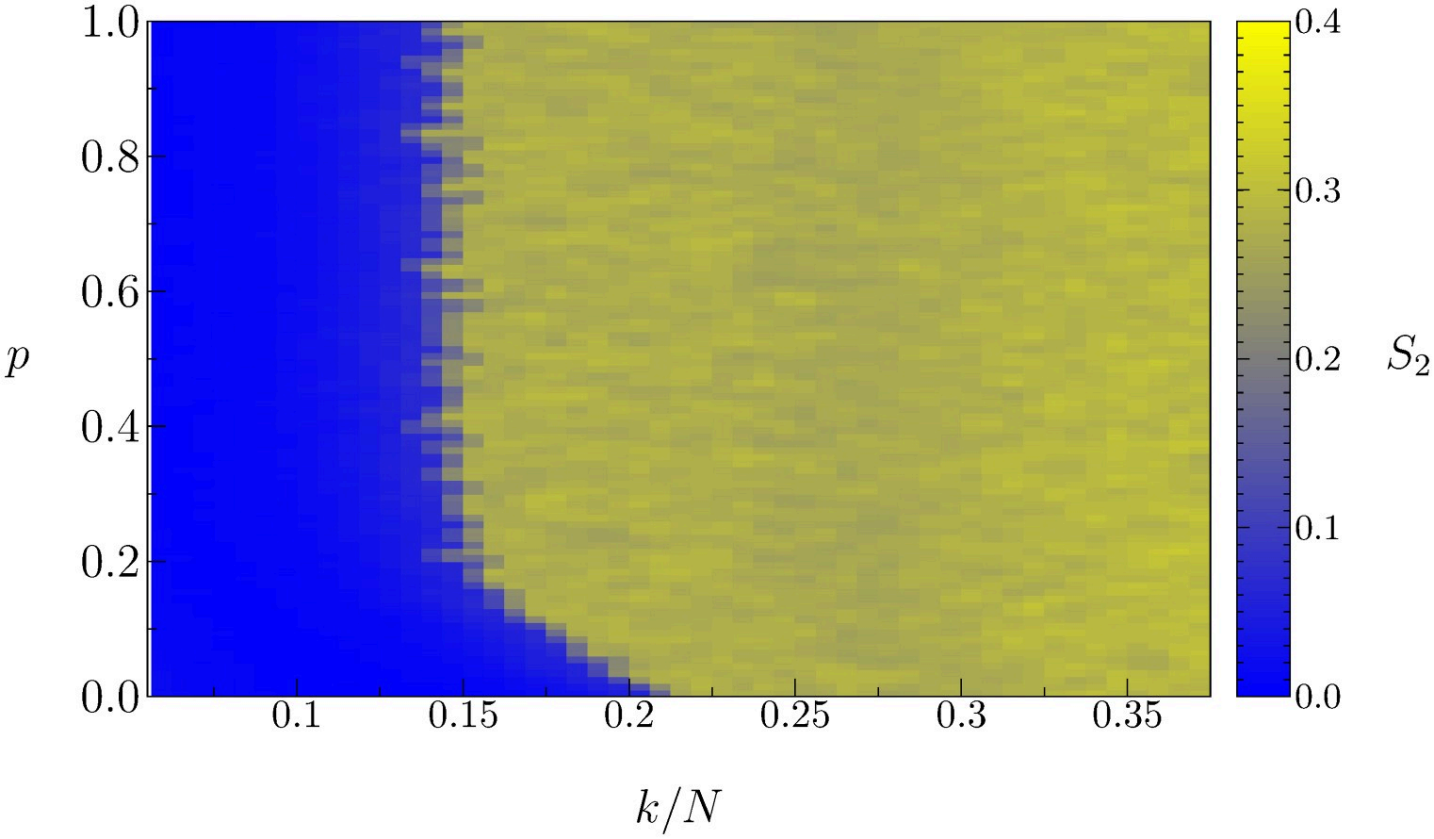
Size of the second largest domain *S*_2_ on the plane of parameters (*p*, *k*/*N*) for a Watts-Strogatz small-world network subject to global mass media trend. The values of *S*_2_ are indicated by a color code on the right. Fixed parameters are *Q* = 0.247, *B* = 0.01, *N* = 800.

Fig 7 shows the size of the second largest domain *S*_2_ calculated on the plane of parameters (*p*, *k*/*N*) for a Watts-Strogatz small-world network. The fully connected network occurs when *k*/*N* → 1. For *p* = 0 this network corresponds to a ring of *N* nodes where each node is connected to *k* neighbors; the size *S*_2_ increases from zero above a critical value of *k*/*N*, as already seen in Fig 6(b). There is a critical curve on this plane that separates the transition from a homogeneous state with *S*_2_ = 0, to a minority growth state of size *S*_2_ > 0.

For increasing values of the rewiring probability, the transition occurs for a constant threshold value *k*/*N* ≈ 0.15. This indicates that the randomness of the connections is not the main network property contributing to the rise of the minority group; rather this effect appears when a critical degree of connections, or range of interactions, is reached. This critical degree is a small fraction of the size of the system. Thus, all-to-all interactions are not essential for the growth of a minority group in a state different from that of the global mass media trend on a network.

**Fig 8:**
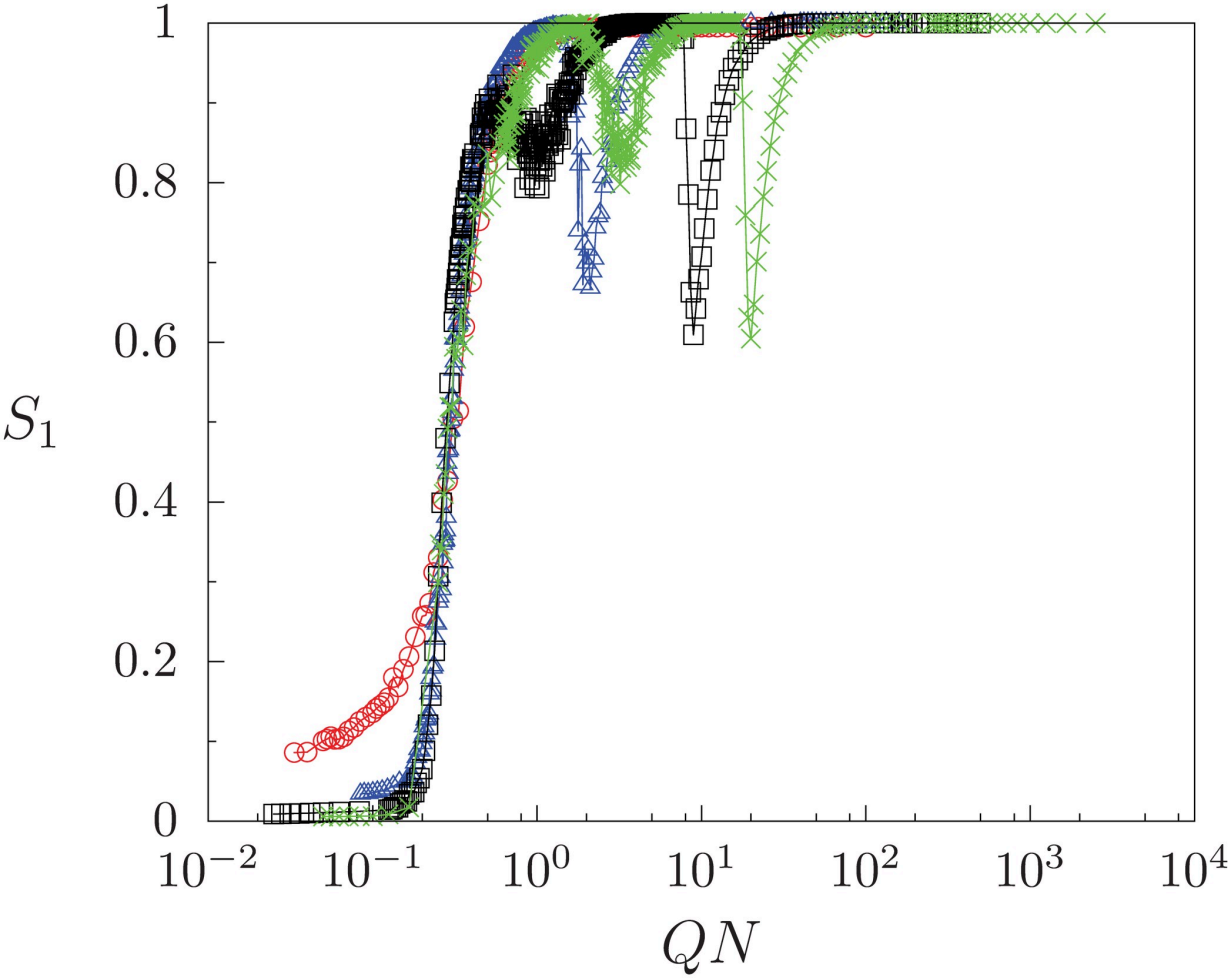
*S*_1_ as a function of *QN* (log scale) with fixed *B* = 0.01 for different sizes of the system: *N* = 200 (circles), *N* = 800 (triangles), *N* = 2500 (squares), and *N* = 5000 (crosses). Each data point is averaged over 100 realizations of initial conditions. Fixed parameter *F* = 10.

Finally, we have explored the behavior of the system for different population sizes *N*. Fig 8 shows the quantity *S*_1_ as a function of *QN* with a fixed mass media intensity *B*, for different values of *N*. The critical point for the transition to phase III scales as *Q*_*c*_ ∼ *N*^−1^, as expected for a fully connected network [33]. We have verified that phases I, II, III, and IV continue to form as *N* increases. However, the drop in size of *S*_1_, and therefore the rise of the largest minority group *S*_2_, are both enhanced with increasing system size.

## 4 Conclusion

We have addressed the question of the competition between the collective self-organization of a globalized society and the influence of endogenous mass media trends, as well as the role of the network connectivity in this competition. The mass media is a global field corresponding to the statistical mode of the states of the social agents. As interaction dynamics, we have considered Axelrod’s rules for the dissemination of culture, a non-equilibrium model whose dynamics allows the existence of non-interacting states. By studying this model on a fully connected network, we have found a phenomenon of spontaneous growth of the largest minority domain in state non-interacting with that of the mass media trend; this domain can occupy almost half of the size of the system. The growth of a largest minority group reflects the tendency towards the spontaneous order related to the agent-agent interactions. We have shown that the competition between the global coupling among the agents and the adaptive nature of the mass media field leads to the repetition of the rise of the largest minority for several values of parameters.

We have characterized four phases for the collective behavior of the system on its space of parameters: (I) an ordered phase; (II) a semi-ordered phase where almost half of the system consists of the largest minority in a state different from that of the mass media; (III) a disordered phase; and (IV) a chimera-like phase where one large domain coexists with many very small domains.

By considering different types of networks such as a random network with varying average degree, a network with varying range of interactions, and a Watts-Strogatz small-world network, we have been able to show that the growth of the largest minority against the global mass media trend is related to the presence of a critical degree of connections in the underlying network. Thus, all-to-all interactions are not necessary for observing this phenomenon.

Our model is connected to the contemporary problem of cultural globalization. Our results shed light on the role that mass media transmitting predominant cultural trends can have on the survival of minority cultural groups in a globalized society. The phenomenon of minority growth in the presence of an autonomous global field should also be expected in other non-equilibrium systems having non-interacting states, such as social and biological systems whose interaction dynamics usually depends on a bound condition and possess long-range interactions. This involves several opinion models as well as models of motile agents such as swarms, bird flocks, fish shoals, bacteria colonies, and non-local interactions in population dynamics.

## Supporting information

S1 File (ZIP)
